# Broadening horizons: microbiota as a novel biomarker and potential treatment for endometriosis

**DOI:** 10.3389/fmicb.2025.1521216

**Published:** 2025-04-17

**Authors:** Min Wang, Wei Liu, Lianwen Zheng, Shuai Ma, Lianhai Jin, Donghai Zhao, Dandan Li

**Affiliations:** ^1^Department of Obstetrics and Gynecology, The Second Hospital of Jilin University, Changchun, China; ^2^College of Laboratory Medicine, Jilin Medical University, Jilin, China; ^3^Low Pressure and Low Oxygen Environment and Health Intervention Innovation Center, Jilin Medical University, Jilin, China; ^4^College of Basic Medicine, Jilin Medical University, Jilin, China

**Keywords:** endometriosis, microbiota, metabolomics, infertility, etiology, biomarkers, therapeutics

## Abstract

As a heterogeneous disease, endometriosis is associated with diagnostic delay. Delayed diagnosis, physical discomfort, hormone therapy, and inconvenience in daily life and work all contribute to a decreased quality of life for endometriosis patients. Early clinical diagnosis is highly important for the intervention and treatment of endometriosis. Currently, reliable non-invasive diagnostic methods are lacking, and laparoscopic examination combined with pathological diagnosis is considered the “gold standard” for definitively diagnosing endometriosis. An increasing number of studies have confirmed the correlation between endometriosis and microbial ecological changes. Microbial dysbiosis is an important factor in the development and progression of endometriosis. Certain key microbial species and their metabolites can induce functional alterations in endometrial cells through various mechanisms, often preceding the emergence of clinical symptoms. Endometriosis are chronic inflammatory diseases, with an immunoinflammatory response as the pathological foundation. The microbiome may participate in the pathological mechanisms of endometriosis through multiple pathways, including mediating inflammatory responses, regulating immune responses, participating in estrogen regulation, interfering with metabolic activities, and modulating the gut–brain axis. Therefore, the microbiome holds potential as an early non-invasive diagnostic and therapeutic target for endometriosis patients. This study summarizes and analyses the correlations between microorganisms and their metabolites and the onset of endometriosis, aiming to provide novel insights into the etiology, diagnosis, and treatment of endometriosis.

## 1 Introduction

Endometriosis refers to the presence, growth, infiltration, and recurrent bleeding of endometrial tissue with growth potential, including glands and stroma, in locations outside the uterine cavity (Taylor et al., 2021; Kvaskoff et al., 2021). Endometriosis predominantly affect adolescent and reproductive-aged women, with clinical pathological classifications encompassing ovarian, peritoneal, deep infiltrating, and other site-specific endometriosis (Horne and Missmer, 2022). Despite being a benign condition, endometriosis exhibit characteristics reminiscent of malignancy, such as implantation, invasion, and distant metastasis, significantly impacting the health and quality of life of women of reproductive age (Arion et al., 2020). The etiology and pathogenesis of endometriosis remain elusive, with surgery or hormone therapy being common therapeutic interventions. However, these approaches are associated with high recurrence rates, adverse effects of hormone therapy, and the risk of postoperative recurrence (Lamceva et al., 2023). With the advancement and maturation of genomics and high-throughput sequencing technologies, the correlation between the human microecological environment and female reproductive health has garnered considerable attention, particularly the relationship between microbial dysbiosis and endometriosis, which has emerged as a current research hotspot (Ventolini et al., 2022). Microbial dysbiosis plays a pivotal role in the initiation and progression of endometriosis (Adnane and Chapwanya, 2022). Imbalances in the microbiome lead to elevated proinflammatory factors and compromised immune function, contributing to the onset of endometriosis (Wang et al., 2022). Over time, immune dysregulation and chronic inflammation create an environment conducive to increased adhesion and angiogenesis, driving a vicious cycle of endometriosis pathogenesis and progression (Zhao et al., 2015). Additionally, intestinal microbial dysbiosis, characterized by an increased abundance of harmful bacteria or decreased levels of beneficial bacteria, may damage the integrity of intestinal epithelial cells and impair the mucosal barrier’s defense against pathogens. This, in turn, can alter central nervous system connections via the gut–brain axis, increasing the expression of inflammatory factors and leading to neuroinflammation and immune dysfunction (Amro et al., 2022). There is a definite correlation between endometriosis and the microbiome, suggesting that the microbiome may play a crucial role in both the pathogenesis and diagnosis of endometriosis.

## 2 Overview of endometriosis

**Figure 1:**
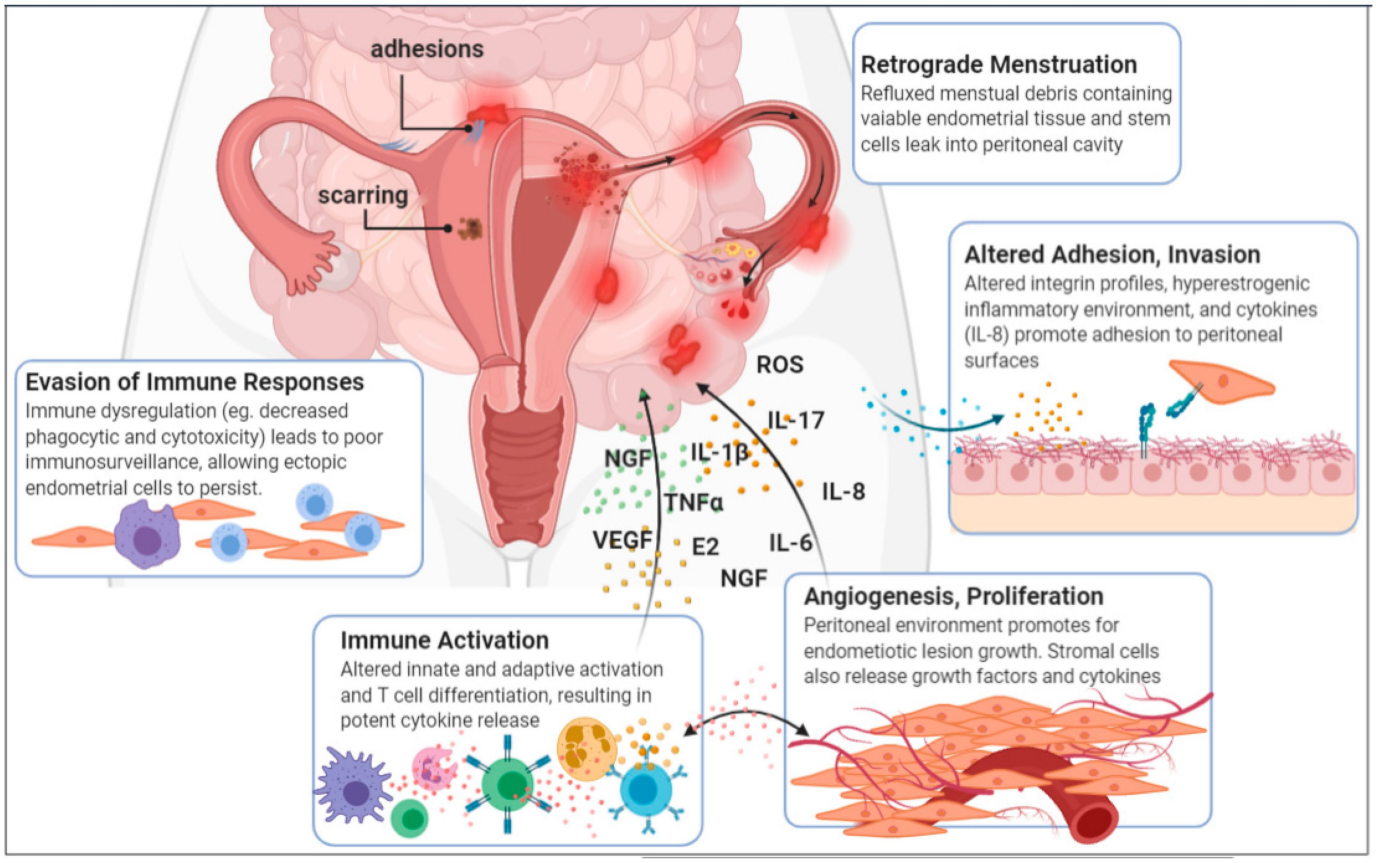
Aetiology and pathogenesis of endometriosis (Jiang et al., 2021).

Endometriosis, a chronic inflammatory and estrogen-dependent gynecological disorder, exhibit extensive pathological manifestations that can affect multiple organ systems (Chapron et al., 2019). The precise pathogenesis of endometriosis remains elusive and is intricately linked to various factors, such as epigenetics, epithelial–mesenchymal transition, inflammation, immunity, and angiogenesis (Franca et al., 2022; Fan and Li, 2022). Clinically, endometriosis present with a wide spectrum of symptoms ranging from asymptomatic to chronic pelvic pain, infertility, dysmenorrhea, and pelvic nodules or masses (Terzic et al., 2021). Owing to the non-specific nature of early symptoms and the lack of definitive serological or imaging diagnostic methods, the rate of missed diagnoses for early-stage endometriosis is alarmingly high (Schenken et al., 1984; Opoien et al., 2013). Delayed diagnosis deprives patients of the opportunity for early medication and timely disease control, often leading to surgical intervention when pelvic masses, severe dysmenorrhea, or infertility arise (He et al., 2022). Even after surgical treatment, endometriosis patients often require adjuvant medication to prevent recurrence (Bedaiwy et al., 2002). Endometriosis significantly impact women’s physical and mental health, as well as their overall quality of life (Cohen Ben-Meir et al., 2022). There is an urgent need for deeper insights into the pathogenesis of endometriosis, the development of precise early non-invasive diagnostic methods, and the search for safer and more effective treatment options (Jiang et al., 2021) (Figure 1). Mounting experimental evidence underscores the notable differences in the microbial communities between endometriosis patients, animal models, and healthy controls.

## 3 Endometriosis accompanied by microbial dysbiosis

The human microecology encompasses a complex and diverse organic ensemble of symbiotic and pathogenic microorganisms residing both within and on the human body. This intricate ecosystem provides an optimal habitat for these microbes, which, in turn, directly or indirectly influence a range of host vital activities (Molina et al., 2020). The microbial communities engage in an intricate interplay with the host, exchanging energy, materials, and genetic information, fostering a dynamic balance of mutual coordination and restraint (Shabbir et al., 2021). Typically, these microbial populations inhabit the oral cavity, gastrointestinal tract, pelvic and abdominal cavities, vagina, and respiratory tract, encompassing bacteria, fungi, eukaryotes, and viruses. The microbiome, on the other hand, refers to the collective genetic material of these microorganisms (Ser et al., 2023; Berg et al., 2020). Research into the correlations between microbiome and their metabolites and endometriosis has emerged as a pivotal focus in etiological studies (Ravel et al., 2011).

### 3.1 Disruption of the gut microbiota

**Figure 2:**
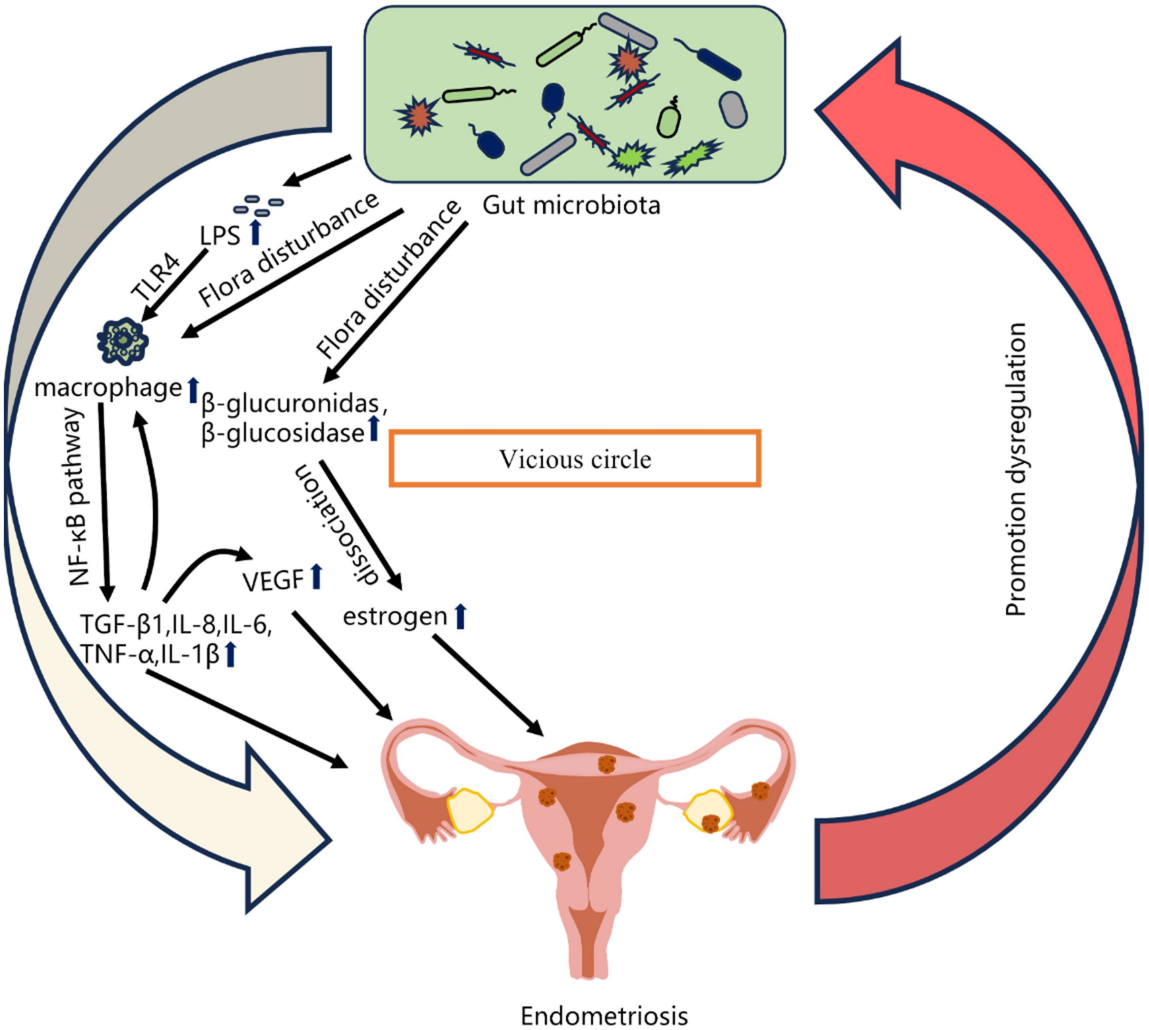
Relationships between gut microbiota imbalance and Endometriosis (Liu et al., 2024).

The gut microbiota represents the largest microbial population in the human body (Aggarwal et al., 2023). There is a close correlation between alterations in the gut microbiome and the onset of endometriosis (Svensson et al., 2021). Imbalances in the gut microbiota compromise the integrity of the intestinal epithelial barrier, increasing gut epithelial permeability and leading to “leakage” of lipopolysaccharides (LPS), ultimately disrupting the endocrine system, immune system, and lipid metabolism (Dicks, 2022). An increased Firmicutes-to-Bacteroidetes (F/B) ratio is considered a key indicator of gut microbial dysbiosis (Trompette et al., 2014). Disturbances in the gut microbiota can cause pelvic inflammatory adhesions, which alter the pelvic anatomy and hinder the transportation of ova and fertilized eggs through the fallopian tubes, contributing to infertility. To elucidate the causal relationship between gut microbes and endometriosis, researchers have established endometriosis animal models. Yuan et al. (2018) reported significant changes in the gut microbiota of endometriosis mice, with increased abundances of Firmicutes and Actinobacteria and decreased Bacteroidetes, suggesting that endometriosis induce gut microbial dysbiosis. Through a comparative analysis of the vaginal, cervical, and gut microbiota between stage III/IV endometriosis patients and healthy controls, Ata et al. (2019) reported increased abundances of potential pathogens, including *Gardnerella*, *Streptococcus*, *Escherichia coli*, *Shigella*, and *Ureaplasma*, in the cervical microbiota of endometriosis patients. The fecal microbiota of endometriosis patients was dominated by *Shigella* and *E. coli*. Studies have also revealed that, compared with healthy individuals, patients with ovarian endometriosis and deep infiltrating endometriosis exhibit significantly decreased abundances of Firmicutes and Clostridia and a notable increase in *Ruminococcus* in their gut microbiota (Shan et al., 2021). These findings point to the potential of the gut microbiota as a novel direction in endometriosis etiology research, and given the ease of sample collection, gut microbes hold promise as non-invasive biomarkers for early endometriosis diagnosis (Leonardi et al., 2020; Liu et al., 2024) (Figure 2).

### 3.2 Dysbiosis of the reproductive tract microbiota

#### 3.2.1 Vaginal microbiota

The vagina serves as a vast microbial chamber (Gao et al., 2022). In healthy females, the vaginal microbiome has a straightforward structure, with *Lactobacillus* species occupying a pivotal and dominant position, accompanied by other bacteria such as *Gardnerella* and *Mycoplasma* (Onywera et al., 2019). Collectively, they maintain the equilibrium of the vaginal microenvironment, preserving vaginal health. The beneficial bacteria within the vagina suppress the growth of harmful microorganisms to a certain extent, inhibiting inflammatory responses and playing a vital role in sustaining normal physiological functions (Liu et al., 2022). *Lactobacillus* secretes acidic substances such as lactic acid and H_2_O_2_, creating an acidic environment in the vagina with a pH that is consistently maintained below 4.5 (Witkin and Linhares, 2017). Lactic acid blocks protein deacetylases, hinders the attachment of other bacteria to epithelial cells, and enhances gene transcription and DNA repair, thereby killing or inhibiting the growth of other bacteria (Wang et al., 2021). When the colonization of *Lactobacillus* decreases, the vaginal pH shifts, facilitating the proliferation of anaerobic bacteria. Detrimental metabolic byproducts, which act on the vagina and cervix, disrupt the protective function of the microecological barrier, are produced or excessively accumulate. This increases the risk of vaginal infections and, consequently, female reproductive system diseases (Mahajan et al., 2022). Notably, in patients with endometriosis, the number of *Lactobacillus* significantly decreases, whereas other harmful bacteria, such as Actinobacteria, particularly *Gardnerella* and *Atopobium*, are significantly enriched in the vaginal microbiota. The imbalance of the vaginal microbiota is closely associated with the development of endometriosis, suggesting that delving into the vaginal microbiome for in-depth etiological research on endometriosis could lead to more effective clinical treatments.

#### 3.2.2 Cervical microbiota

The richness and diversity of the cervical microbiome in endometriosis patients are significantly reduced (Kitaya and Yasuo, 2023). Yang et al. (2023) reported that the cervical-to-vaginal microbial ratios of certain genera are correlated with the severity of endometriosis and that these genera are clearly associated with retrograde infection pathways. Akiyama et al. (2019) reported increased levels of Enterobacteriaceae and *Streptococcus* in the cervical mucus of women undergoing epithelial–mesenchymal transition, where impaired reproductive tract immunity allows pathogens to persist in the cervical mucus, suggesting that bacteria may ascend into the uterine cavity to induce inflammatory responses. Research has revealed not only that the cervical microbiome of endometriosis patients differs from that of healthy women but also that there are some differences in cervical microbial populations between endometriosis patients at stages I/II and III/IV, indicating that the structure of the cervical microbiome may be dynamic during disease progression. These differential microbial populations could serve as biomarkers for endometriosis staging (Chang et al., 2022). The decreased richness and diversity of the cervical microbiota are linked to the severity of endometriosis, including higher CA125 levels and more severe symptoms such as dysmenorrhea and infertility. A systematic review encompassing 28 clinical studies and 6 animal experiments also indicated that an increase in bacterial vaginosis-associated pathogens and a depletion of *Lactobacillus* in the cervical-vaginal microbiome are associated with endometriosis and infertility (Salliss et al., 2021). Detection of the cervical microbiota may be biased by the influence of the vaginal microbiota during sampling, and there are also subjective differences in research techniques and data analysis.

#### 3.2.3 Uterine microbiota

Compared with the cervical and vaginal microbiota, the endometrial microbiota of healthy women has a lower biomass but greater diversity and a notably distinct microbial composition, dominated by *Lactobacillus*, *Prevotella*, *Streptomyces*, and other species (Medina-Bastidas et al., 2022). Wessels et al. (2021) performed endometrial biopsies on 12 endometriosis patients and 9 non-endometriosis patients and reported a relatively high Shannon diversity index in the microbiota of endometriosis patients. Wei et al. (2020) reported that *Pseudomonas* and *Acinetobacter* were the hallmark strains in the uterine cavity of endometriosis patients, with the species richness of bacterial colonies gradually increasing from the vagina to the upper genital tract. Sampling analysis of women undergoing laparoscopic surgery revealed an association between the vaginal microbiota and endometriosis, with a greater abundance of infectious bacteria in the vagina of endometriosis women than in non-endometriosis women. Furthermore, a strong correlation was noted between *Lactobacillus* in both the vaginal and endometrial microbiomes (Oishi et al., 2022). Khan et al. (2014, 2016) reported that, compared with non-endometriosis patients, endometriosis patients presented a significant decrease in the proportion of Lactobacillaceae in the uterine cavity, accompanied by a marked increase in the ratios of Streptomycetaceae, Staphylococcaceae, and Enterobacteriaceae. Given the inherent difficulty for patients to accept uterine cavity sampling, resulting in limited clinical samples and insufficient research, researchers need to continue collecting data to explore the patterns and characteristics of the endometrial microbiota in endometriosis. Pelzer et al. (2018) reported that anaerobes and staphylococci are the most abundant members of the fallopian tube microbiota and that there are differences in the microbial communities between the left and right fallopian tubes, as well as between the interstitial and ampullary portions, which may be related to the impact of endometriosis lesions on pelvic anatomy and retrograde menstrual flow.

### 3.3 Dysbiosis of the peritoneal microbiota

The pelvic and abdominal cavities are the primary sites of endometriosis pathogenesis. In females, the pelvic and abdominal cavities communicate with the uterine cavity, allowing the dissemination of the uterine microbiota into these areas during uterine peristalsis and contractions (Yuan et al., 2022). Changes in the gut microbiota composition induced by endometriosis lead to intestinal cell apoptosis, mucus degradation, and disruption of tight junction protein complexes, thereby increasing intestinal wall permeability (Young et al., 2017), which can also result in the translocation of many Gram-negative bacteria outside the intestinal lumen (Mazur-Bialy et al., 2017). Lee et al. (2021) reported that the abundances of *Acinetobacter*, *Pseudomonas*, *Streptococcus*, and *Enhydrobacter* were significantly elevated in the peritoneal washings of endometriosis patients, whereas the abundances of *Propionibacterium* and *Actinomyces* were markedly decreased. Additionally, endometriosis patients presented significantly increased levels of IL-6, IL-10, IL-13, and TNFα in their peritoneal fluid, along with increased epithelial permeability, which allowed the release of gastrointestinal and urogenital microbiota into the peritoneal environment. Consequently, the microbial composition in the peritoneal environment of endometriosis patients also underwent alterations, dominated by Proteobacteria and Firmicutes, followed by Actinobacteria, Bacteroidetes, and Clostridia. Hernandes et al. (2020), in their study of the microbiota in peritoneal fluid from patients with deep endometriosis lesions, reported significantly increased abundances of *Alishewanella*, *Enterococcus*, and *Pseudomonas*, with a scarcity of *Lactobacillus*.

### 3.4 Dysbiosis of the oral microbiota

As a component of the human microbiome, the oral microecology of endometriosis patients reveals *Streptococcus*, *Haemophilus parasuis*, and *Prevotella* as the most prevalent genera in saliva (Hu et al., 2023; Hicks et al., 2025). While no direct correlation between oral microecology and endometriosis has been established thus far, oral microbial imbalance has been linked to certain inflammatory conditions. Following mild brain injury, there is a reduction in species richness and community diversity within the oral microbiota, with Proteobacteria, Bacteroidetes, Firmicutes, and Actinobacteria being the most representative and abundant phyla. Notably, there is a marked increase in Proteobacteria and Bacteroidetes over time following brain injury (Wang et al., 2023). Atherosclerosis fundamentally involves endothelial cell damage, and periodontal infections can either directly or indirectly contribute to immune dysregulation and increased production of systemic inflammatory mediators, thereby exacerbating atherosclerosis (Slocum et al., 2016).

## 4 Mechanisms by which the microbiota affects endometriosis

### 4.1 Microbiota-mediated inflammatory reactions contribute to the onset and progression of endometriosis

Inflammation is a pivotal process in the progression of endometriosis. In endometriosis, microecological imbalances within the gut microbiome trigger intestinal inflammation, leading to dysregulation of the immune response and the establishment of an immunosuppressive environment. This environment enables escaped endometrial cells to proliferate and grow outside the uterus, resulting in symptoms such as pain, tissue remodeling, fibrosis, adhesions, and infertility (Salmeri et al., 2024). Bailey and Coe (2002) reported a significant decrease in *Lactobacillus* counts and an increase in Gram-negative aerobic and facultative anaerobic bacteria in the feces of rhesus monkeys with endometriosis compared with healthy controls. Additionally, the incidence of intestinal inflammation was notably greater in endometriosis-affected monkeys than in control monkeys, suggesting that changes in the gut microbiota may be influenced by endometriosis-related intestinal inflammation (Bailey and Coe, 2002). Periodic bleeding from endometriosis provides a nutrient-rich environment for microorganisms, exacerbating pelvic inflammation and disease progression (Baker et al., 2017). Agostinis et al. (2020) demonstrated reduced T lymphocyte activity and expanded populations of macrophages, eosinophils, and neutrophils in endometriosis patients. Furthermore, abnormal activation of the complement system was detected in the peritoneal cavity of women with endometriosis. The regurgitated menstrual blood and peritoneal fluid of endometriosis patients contain relatively high levels of *E. coli*, whose endotoxins and LPS, as primary inflammatory mediators, are recognized and bound by Toll-like receptor 4 on macrophages and other immune cells. This interaction triggers the rapid secretion and release of secondary inflammatory mediators, fostering a pelvic inflammatory milieu that promotes inflammatory infiltration, hyperplasia, and angiogenesis in endometriosis (Khan et al., 2010).

### 4.2 Microbial ecology modulation of immune responses contributes to the onset and progression of endometriosis

Endometriosis is a disease characterized by immune response dysregulation, featuring perturbed polarization of pelvic macrophages. These macrophages aid in evading immune surveillance by ectopic endometrial cells, thereby facilitating the onset and progression of endometriosis (Khan et al., 2019). Campos et al. (2018) revealed that *Mycoplasma genitalium* can increase interferon-*γ* and interleukin-1β levels, modulating the local immune response in endometriosis patients. Studies have shown that endometriosis patients exhibit immune deficiencies and alterations in invasion within the endometrial microenvironment. LPS, through Toll-like receptor 4 signaling, is a significant factor contributing to epithelial–mesenchymal transition, a crucial process in endometriosis pathogenesis (Khan et al., 2018). The epithelial–mesenchymal transition plays a pivotal role in the ability of ectopic endometrial cells to successfully invade extrauterine organs and tissues, possibly related to its ability to diminish immune clearance mechanisms (Xu et al., 2020). Microbial dysbiosis impairs the immune clearance function of ectopic endometrial cells, leading to increased LPS levels that continuously activate the programmed death-1 (PD-1) pathway. This activation results in the overexpression of programmed death-1 and programmed death-ligand 1, causing T-cell exhaustion and immune tolerance. Consequently, the body’s immune defenses weaken, promoting infiltration of ectopic lesions, adhesions, and angiogenesis within the endometrial tissue, thus accelerating endometriosis progression (Feng et al., 2023; Santoso et al., 2020).

### 4.3 The influence of the microbiota on endometriosis through metabolic pathways

#### 4.3.1 Increased estrogen metabolism

**Figure 3:**
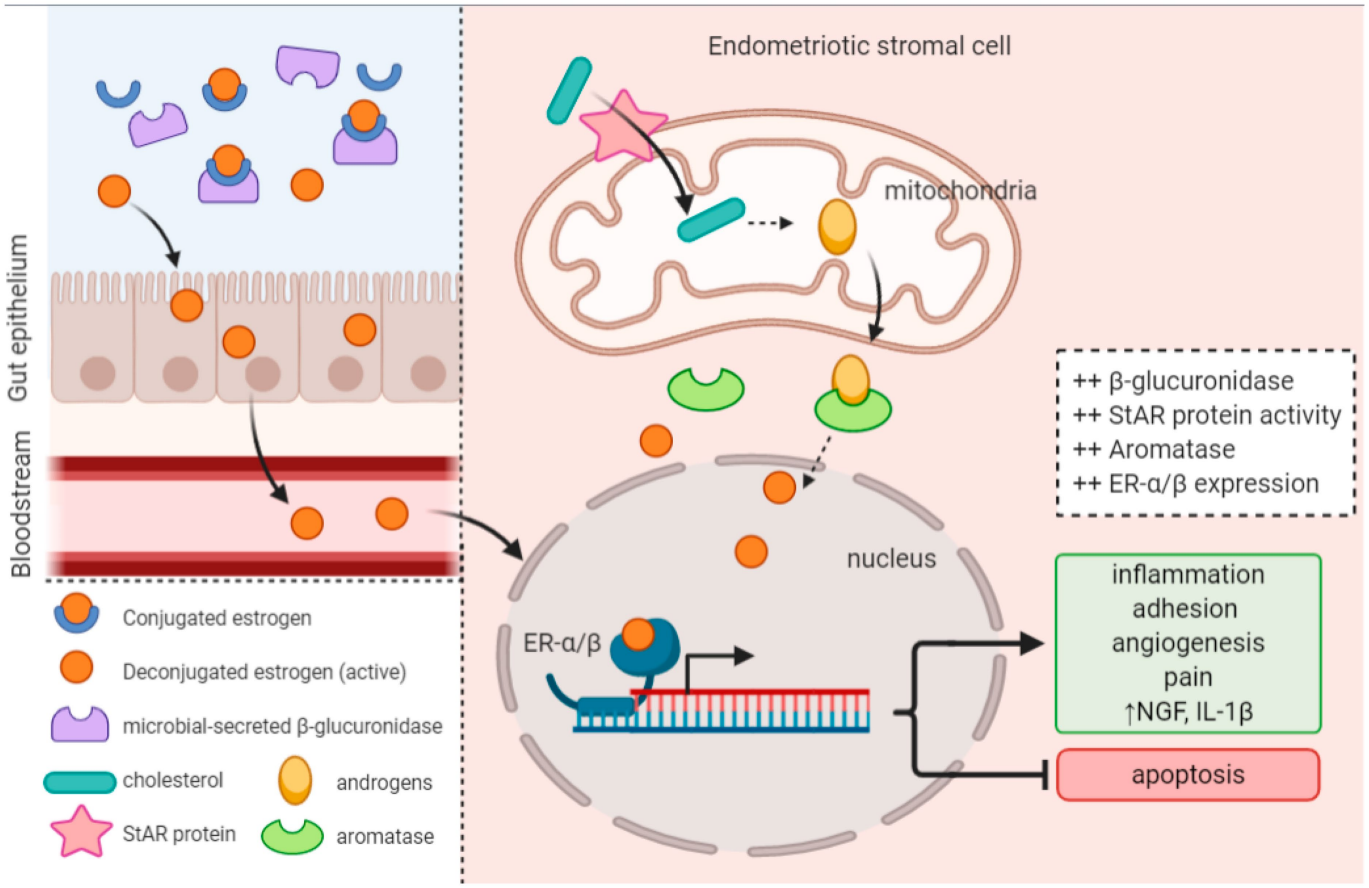
Altered estrobolome activity and upregulated enzyme expression produce a hyperestrogenic environment that promotes endometriosis onset and progression (Jiang et al., 2021).

The gut microbiota microecology participates in the initiation and progression of endometriosis by modulating circulating estrogen levels (Laschke and Menger, 2016; Lu et al., 2017) (Figure 3). Baker et al. (2017) discovered that the gut microbiota is involved in intestinal estrogen metabolism, which is specifically related to β-glucuronidase, an enzyme that can deconjugate estrogens into their active forms. In cases of gut dysbiosis, when the Firmicutes/Bacteroidetes (F/B) ratio decreases, the secretion of intestinal β-glucuronidase increases, leading to elevated estrogen levels. These active estrogens can subsequently travel via the bloodstream to distant mucosal sites, including the endometrium (Wei et al., 2023). The collective genes of microorganisms capable of metabolizing estrogens within the gut are termed “estrobolomes,” which primarily encompass *Bacteroides*, Bifidobacteria, *Escherichia coli*, and *Lactobacillus* (Flores et al., 2012). In endometriosis research, the estrobolome hypothesis connects the crosstalk mechanism among endometriosis, estrogens, and microorganisms (Pai et al., 2023). Studies on the gut microbiota of endometriosis patients have identified more than 10 bacterial species whose intestinal profiles significantly differ. Additionally, these patients exhibit notably elevated plasma concentrations of estradiol and IL-8, along with an imbalanced ratio of Bacteroidetes to Firmicutes. The enrichment of Erysipelotrichaceae in fecal samples from endometriosis patients and significantly increased levels of estriol, 16-epiestriol, 16*α*-hydroxyestrone, and 2-methoxyestradiol have also been reported. Research has further indicated a correlation between the composition of the vaginal microbiota and age, with the 40–50 age group being particularly prone to dysbiosis. This age range coincides with the gradual decline in ovarian function and estrogen levels in women, suggesting a link between microorganisms and estrogen metabolism.

#### 4.3.2 Abnormal lipid metabolism

Analysis of KEGG pathways associated with the gut microbiota of endometriosis patients suggested enhanced tryptophan metabolism, α-linolenic acid metabolism, and ether lipid metabolism. Similarly, in endometriosis mouse models, abnormally expressed lipids such as phosphatidylcholine, sphingomyelin, phosphatidylethanolamine, and triglycerides were identified, with triglycerides closely linked to peritoneal inflammation. Ni et al. (2020) reported increased levels of chenodeoxycholic acid and ursodeoxycholic acid and a decreased abundance of α-linolenic acid in endometriosis mice. Exogenous supplementation with α-linolenic acid restored the abundance of Firmicutes and Bacteroidetes gut dysbiosis, reduced macrophage aggregation, and improved intestinal barrier function and the inflammatory environment in the abdominal cavity. Matta et al. (2022) reported imbalances in bile acid homeostasis and lipase activity in endometriosis patients, wherein disruptions in bile acid synthesis and metabolism can compromise intestinal barriers, fostering bacterial overgrowth and facilitating LPS entry into the bloodstream, thereby activating aberrant inflammatory responses. A study reported decreased abundances of Firmicutes taxa, such as Lachnospiraceae and Ruminococcaceae, in endometriosis patients, which normally produce short-chain fatty acids to modulate host physiology and immune function (Li et al., 2022). Notably, endometriosis mice presented significantly reduced concentrations of butyrate, isobutyrate, and valerate, with butyrate inhibiting ectopic endometrial growth and inflammatory cell infiltration. Butyrate suppresses the survival and lesion growth of human endometriotic cells through G protein-coupled receptors, histone deacetylases, and the GTPase-activating protein RAP1, thereby inhibiting human endometriosis.

### 4.4 Brain–gut axis

**Figure 4:**
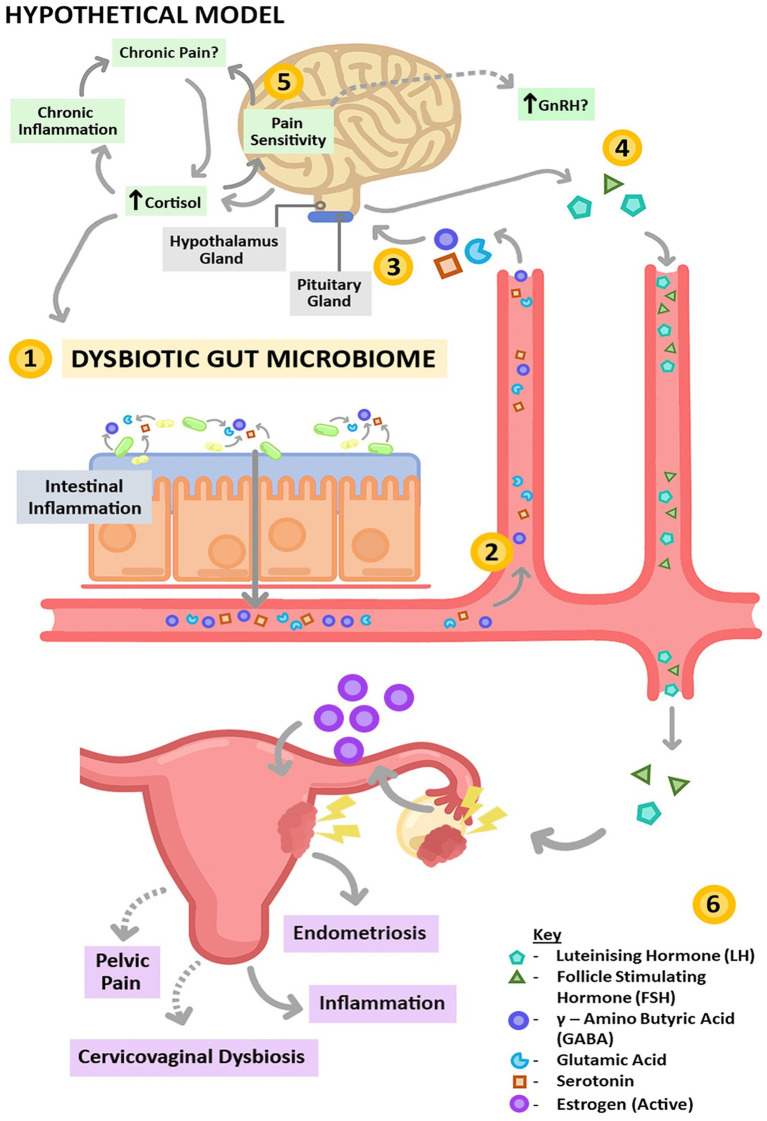
Brain–gut axis. Changes in neuroactive compound/protein production caused by gut dysbiosis can increase the level of GnRH (Salliss et al., 2021).

The brain–gut axis encompasses the intricate interplay between the gut microbiota, immune system, neuroendocrine system, and central nervous system (Mayer et al., 2022; Kesika et al., 2021). Disruptions in the gut microbiome can lead to elevated levels of neuroactive metabolites such as glutamate and gamma-aminobutyric acid, which stimulate brain neurons and stimulate estrogen secretion from the ovaries via the H-P-O axis (Liao et al., 2021). In turn, estrogen promotes nerve growth and differentiation, influencing pain perception through various mechanisms. Imbalances in the gut microbiota and disruptions in the brain–gut axis contribute to various types of pain (Yano et al., 2019). Research suggests that the gut microbiota may enhance central sensitization associated with chronic pain in endometriosis by modulating the activities of microglia, astrocytes, and immune cells, thereby intensifying patients’ pain perception (Ding et al., 2022). Moreover, gut microbiota dysbiosis can lead to neuronal death through oxidative stress and neuroinflammation, altering cognitive and immune functions in the body and accelerating the progression of endometriosis (Hu et al., 2023) (Figure 4).

## 5 Clinical significance and future directions of the use of the microbiota in endometriosis

### 5.1 Biomarkers for non-invasive diagnosis

The search for reliable early, non-invasive biomarkers for biological diagnosis has emerged as a pivotal research focus in endometriosis studies (Uzuner et al., 2023). Perrotta et al. (2020) conducted an analysis of vaginal secretions and fecal samples from endometriosis patients and healthy controls during the follicular and menstrual phases and revealed that the *Anaerococcus* genus within the vaginal microbiome can potentially predict the stage of endometriosis (I/II or III/IV). Chen et al. (2020) developed a model based on the female genital tract microbiome, which can differentiate between infertility caused by endometriosis and other factors, suggesting that the vaginal or cervical microbiome may aid in detecting common upper genital tract diseases. Through screening and analysis of paired samples from feces, cervical mucus, and peritoneal fluid, *Ruminococcus* in the gut and *Pseudomonas* in the pelvic cavity were identified as potential biomarkers. A disease prediction model constructed on the basis of the core microbiota indicated that the gut microbiota outperforms the cervical mucus microbiota in the early diagnosis of endometriosis. Furthermore, the combination of the vaginal microbiota with CA125 offers a novel approach for discriminating endometriosis-related pelvic pain. Elevated serum irisin levels and reduced expression of the visfatin and angiogenin genes in endometriosis patients have been proposed as potential biomarkers for endometriosis (Kaya Sezginer et al., 2022; Slate-Romano et al., 2022). These studies underscore the potential of the microbiota as a crucial tool for early, non-invasive diagnosis or screening of endometriosis (Huang et al., 2021).

### 5.2 Potential approaches for microbiome-based therapies in endometriosis

#### 5.2.1 Diet adjustment

The correlation between diet and the onset of endometriosis has been well established (Parazzini et al., 2013). Dietary interventions can impact the pathogenesis of endometriosis by modulating microbial-mediated metabolic alterations, reducing the release of proinflammatory factors, angiogenesis, and cell proliferation (Tomio et al., 2013). Women with high intakes of fruits, vegetables, dairy products, and fish and nuts rich in polyunsaturated fatty acids (PUFAs) have a decreased risk of developing endometriosis (Fu et al., 2021); conversely, increased consumption of products rich in trans fats, red meat, and alcohol is associated with an elevated risk of endometriosis (Missmer et al., 2010). The anti-inflammatory effects of diets high in *ω*-3 PUFAs, which are achieved by reducing TNF-*α* and IL-6 levels, contribute to lowering the risk of endometriosis among women (Yamamoto et al., 2018). This protective effect has also been validated in endometriosis animal models, where diets rich in omega-3 fatty acids and polyunsaturated fatty acids (PUFAs) exhibit anti-inflammatory properties and suppress endometriosis lesions (Attaman et al., 2014). Studies indicate that low-fat diets can increase the α diversity of the gut microbiota, whereas high-fat diets are linked to a reduction in *Bacteroides* and *Coprobacillus* species. These diet-induced alterations in the gut microbiota offer novel insights into the development of therapeutic strategies targeting gut bacterial metabolites for endometriosis treatment (Parpex et al., 2024).

#### 5.2.2 Probiotics

Probiotics, as live microorganisms, can modulate the composition of the gut microbiota, thereby influencing β-glucuronidase activity within the body and subsequently regulating estrogen levels. Additionally, they contribute to maintaining immune system homeostasis by promoting anti-inflammatory responses and modulating immune cell activities, which in turn reduces the generation of inflammatory environments associated with endometriosis (You et al., 2022; Markowiak and Slizewska, 2017). Itoh et al. (2011a,b) reported that *Lactobacillus gasseri* OLL2809 inhibits the development of ectopic endometrial cells in the peritoneal cavity by activating NK cells. Kaczmarczyk et al. (2022) noted significant differences in the levels of IL-1 and IL-6 produced by peripheral blood mononuclear cells between endometriosis patients and healthy controls, whereas *Lactobacillus acidophilus* was found to induce the production of these cytokines. Furthermore, probiotics enhance neurotransmitter synthesis and signaling within the gut microbiota. By regulating neurotransmitter levels, they may influence pain pathways in endometriosis patients, mitigating pain perception and consequently affecting VAS scores. This modulation is attributed primarily to the regulation of immune responses involving IL-12 and NK cells. Research has shown that exogenous supplementation with the microbial metabolite unsaturated fatty acid alpha-linolenic acid improves the gut microbiota structure, dominant bacterial abundance, and intestinal barrier function in endometriosis mice. This, in turn, regulates intraperitoneal LPS levels and the inflammatory environment, thereby ameliorating endometriosis symptoms (Ni et al., 2021). Other studies have reported that oral *Lactobacillus* administration is beneficial for reducing the severity of dysmenorrhea, dyspareunia, and chronic pelvic pain among endometriosis patients (Ustianowska et al., 2022).

#### 5.2.3 Microbial transplantation and antiestrogen therapy

**Figure 5:**
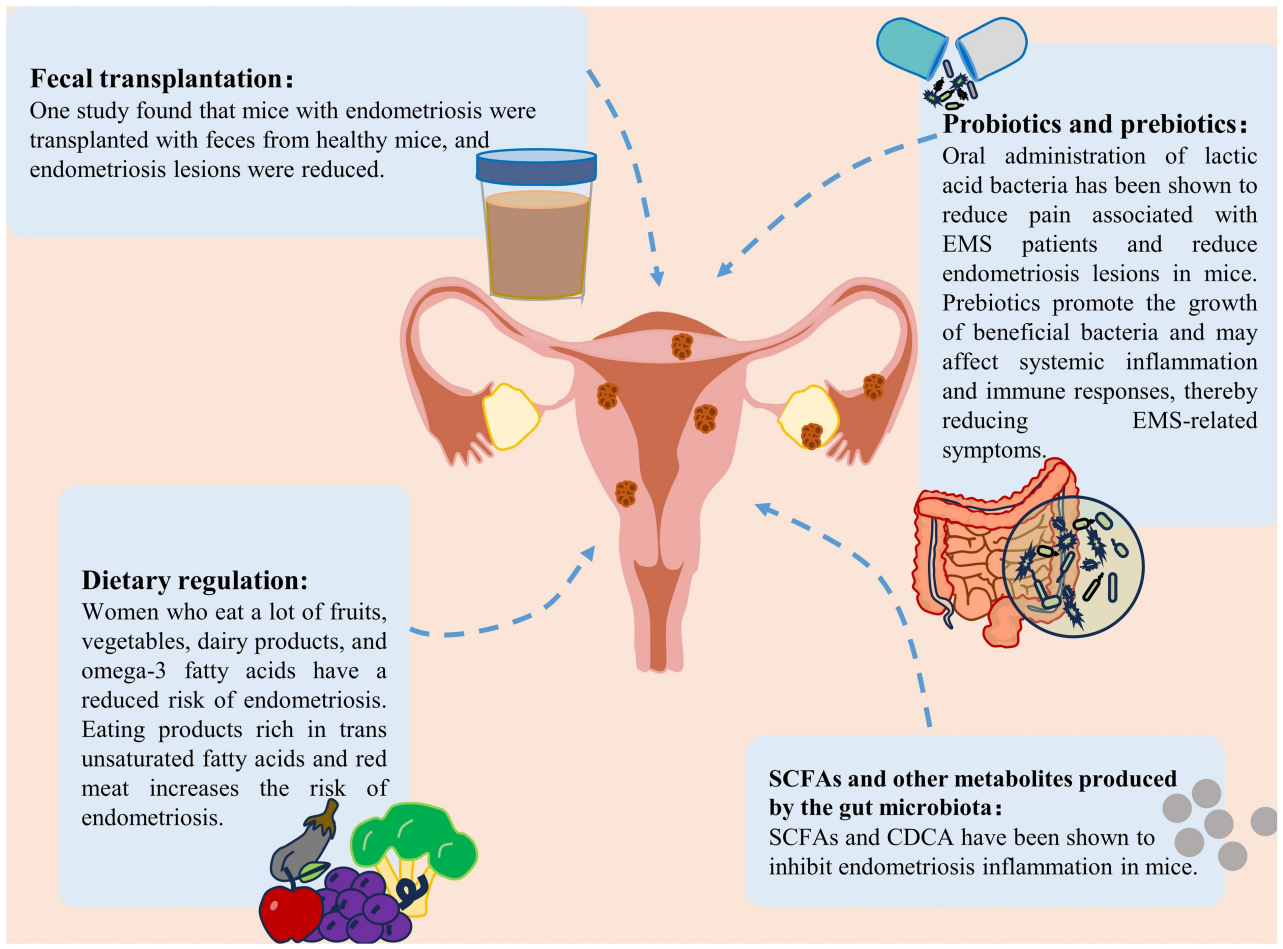
Treatment strategies for endometriosis. Treatment strategies for endometriosis include the use of probiotics and prebiotics, fecal bacterial transplantation, gut microbiota metabolite supplementation and dietary modification (Liu et al., 2024).

Microbiota transplantation holds promise as a potential tool for future endometriosis treatment (Li et al., 2023; Talwar et al., 2025). Chadchan et al. (2019, 2023) reported that mice with gut microbiota depletion, achieved through broad-spectrum antibiotics or metronidazole treatment, presented significantly reduced endometriosis lesions and alleviated inflammation. When these mice were orally gavaged with feces from endometriosis, their endometriosis lesions and inflammation were restored. In contrast, gavage with feces from healthy mice failed to restore endometriosis lesions (Chadchan et al., 2023; Chadchan et al., 2019). Lu et al. (2022) compared the effects of vaginal microbiota transplantation and gonadotropin-releasing hormone agonist treatment in mice with endometriosis and reported that both approaches had nearly identical inhibitory effects on endometriosis lesion growth, suggesting that the vaginal microbiota can promote endometriosis development. While microbiota transplantation is a promising approach, challenges remain in standardizing the isolation, formulation, dosage, and timing of microbial administration to ensure optimal microbiota transplantation and maintenance for effective clinical responses (Takagi et al., 2019). Studies have shown that antiestrogen therapy increases the *α*-diversity of gut bacteria in endometriosis patients, and a positive correlation exists between gut bacterial species and urinary estrogen and its metabolite concentrations in endometriosis patients, indicating a link between the microbiota and estrogen metabolism in endometriosis (Le et al., 2021). Currently, one of the mechanisms for treating endometriosis involves inhibiting the H–P–O axis or antagonizing estrogen with progestins to reduce estrogen levels and suppress lesion growth (Rzewuska et al., 2023). However, these drugs are associated with side effects such as hypoestrogenism symptoms, irregular vaginal bleeding, and breast stimulation. If the mechanisms underlying the influence of the microbiota on estrogen metabolism can be elucidated, it may pave the way for the development of novel endometriosis treatment strategies (Quaranta et al., 2019) (Figure 5).

## 6 Conclusion

The human microbiome is intricately linked with endometriosis (Saunders and Horne, 2021; Tang et al., 2024) (Table 1). The microbiota can drive the onset and progression of endometriosis by modulating inflammatory responses, estrogen dynamics, metabolism, immune system function, and oxidative stress and increasing angiogenesis (Guo et al., 2024). Patients with endometriosis exhibit microbial imbalances in various body sites, which are strongly correlated with the pathogenesis of endometriosis. Conversely, the occurrence of endometriosis exacerbates microbial dysbiosis, creating a vicious cycle that accelerates disease progression. Breaking this cycle may yield breakthroughs in the diagnosis and treatment of endometriosis. Modulating microbial homeostasis through diet, medications, probiotics, or microbiota transplantation represents potential therapeutic avenues for endometriosis. Current research on the endometriosis microbiome has focused primarily on the gut microbiota because of its accessibility, diversity, and abundance. However, considering the unique physiological and pathological features of females, the cervical and vaginal microbiota, which are also representative, specific, and easily accessible to diverse microbial populations, merit further investigation. The discovery of microbiome-related biomarkers in the future could facilitate early, non-invasive diagnosis of endometriosis, thereby reducing delayed diagnoses. These findings have significant implications and vast potential for the prevention, diagnosis, and treatment of endometriosis.

**Table 1:** A summary of recent literature on the microbiota and endometriosis.

Study	Species type	Sample	Technic	Conclusion
Svensson et al. (2021)	Clinical patients	Fecal pellets	16s rRNA sequencing	*Prevotella* has been associated with constipation, flatulence, bloating, vomiting and nausea in endometriosis. High abundance of *Lactococcus*, lower abundance of *Odoribacter* and higher abundance of *Prevotella* in endometriosis.
Khan et al. (2010)	Clinical patients	Menstrual blood and peritoneal fluid	ELISA of macrophages from peritoneal fluid and epithelial/stromal cells from biopsy specimens	Menstrual blood of people with endometriosis has a higher concentration of *E. coli* compared to healthy controls.
Khan et al. (2016)	Clinical patients	Endometrial swabs and cystic fluid	16s rRNA sequencing	Abundance of Streptococcaceae, Staphylococcaceae, Enterobacteriaceae and lowered Lactobacillae in GnRHa treated women.
Akiyama et al. (2019)	Clinical patients	Cervical Mucus	16s rRNA sequencing	Enterobacteriaceae and *Streptococcus* were more commonly found in women with endometriosis.
Ata et al. (2019)	Clinical patients	Stool sample Vaginal and endocervical swabs	16s rRNA sequencing	Absence of *Atopobium* in vaginal and cervical microbiota. Increased *Gardnerella* in cervical microbiota. Dominant gut microbiota in endometriosis group—*Escherichia* and *Shigella*. Predominant population of lower genital tract—*Lactobacillus*. *Alloprevotella* significantly decreased in the cervix.
Chen et al. (2020)	Clinical patients	Cervical swabs and posterior fornix swabs	16s rRNA sequencing	Higher prevalence of *Atopobium* in endometriosis-adenomyosis group.
Hernandes et al. (2020)	Clinical patients	Vaginal fluid, eutopic endometrium, and endometrial lesion tissue samples	16s rRNA sequencing	Vaginal fluid, eutopic endometrium, and endometriotic lesions: similar profiles in microorganisms but higher abundance of *Lactobacillus*, *Gardnerella*, *Streptococcus*, and *Prevotella*. Deep endometriosis lesions: less *Lactobacillus* and higher abundance of *Alishewanella*, *Enterococcus*, and *Pseudomonas*.
Wei et al. (2020)	Clinical patients	Lower reproductive tract swabs: lower third of the vagina, posterior vaginal fornix, cervical mucus. Upper reproductive tract samples: surgery	16s rRNA sequencing	Shift in microbiota distribution starting from the cervical samples and progressively increasing upward the reproductive tract, decreased *Lactobacillus* in lower tract, enriched Sphingobium sp. and *Pseudomonas viridiflava* in endometrium and peritoneal fluid.
Khan et al. (2021)	Clinical patients	Endometrial samples	16s rRNA sequencing	Decreased *Gardnerella*, *Prevotella*, *Acetobacter*, *Atopobium*, *Megasphaera*, and *Bradyrhizobium* in patients with endometriosis in treatment with LVFX or LVFX + GnRHa, reduced occurrence rate of chronic endometritis after GnRHa + LVFX treatment comparison to GnRHa treatment group.
Ni et al. (2020)	C57BL/6J mice	Feces from cecum	16s rRNA sequencing	The abnormal fecal metabolites, particularly those related to secondary bile acid biosynthesis and the alpha-linolenic acid pathways, influenced by dysbiosis, may serve as distinctive features in mice with endometriosis and as potential markers for distinguishing the disease.
Cao et al. (2020)	Sprague Dawley rats	Fecal pellets	16s rRNA sequencing	Letrozole and SFZYD reduce the inflammatory response in both ectopic and eutopic endometrial tissues, which could be associated with the decrease in the Firmicutes/Bacteroidetes ratio.
Yuan et al. (2018)	C57BL/6J mice	Fecal pellets	16s rRNA sequencing	Firmicutes/Bacteroidetes ratio was elevated in mice with endometriosis, indicating that endometriosis may induce dysbiosis.
Chadchan et al. (2019)	C57BL/6 mice	Fecal pellets, peritoneal fluid, endometriotic lesions	16s rRNA sequencing	Administering fecal material from mice with endometriosis via oral gavage restored both the growth of endometriotic lesions and inflammation.
Bailey and Coe (2002)	Female rhesus monkeys	Fecal samples	Coproculture	Decreased lactobacilli and increased gram-negative anaerobes and facultative anaerobes.
Huang et al. (2021)	Clinical patients	Feces, cervical mucus, and peritoneal fluid from patients with endometriosis	16s rRNA sequencing	Genera of *Ruminococcus* and *Pseudomonas* were identified as potential biomarkers in gut and peritoneal fluid.
Wei et al. (2023)	C57BL/6 mice	Fecal pellets	16s rRNA sequencing	β-glucuronidase promoted EMs development directly or indirectly by causing macrophage dysfunction.
